# The Role of the Receptor for Advanced Glycation End-Products in a Murine Model of Silicosis

**DOI:** 10.1371/journal.pone.0009604

**Published:** 2010-03-19

**Authors:** Lasse Ramsgaard, Judson M. Englert, Jacob Tobolewski, Lauren Tomai, Cheryl L. Fattman, Adriana S. Leme, A. Murat Kaynar, Steven D. Shapiro, Jan J. Enghild, Tim D. Oury

**Affiliations:** 1 Department of Pathology, University of Pittsburgh School of Medicine, Pittsburgh, Pennsylvania, United States of America; 2 Department of Molecular Biology, Center for Insoluble Protein Structures (inSPIN) and Interdisciplinary Nanoscience Center (iNANO), University of Aarhus, Aarhus, Denmark; 3 Department of Environmental and Occupational Health, University of Pittsburgh, Pittsburgh, Pennsylvania, United States of America; 4 Department of Medicine, University of Pittsburgh School of Medicine, Pittsburgh, Pennsylvania, United States of America; 5 Departments of Critical Care Medicine and Anesthesiology, University of Pittsburgh School of Medicine, Pittsburgh, Pennsylvania, United States of America; University of Giessen Lung Center, Germany

## Abstract

**Background:**

The role of the receptor for advanced glycation end-products (RAGE) has been shown to differ in two different mouse models of asbestos and bleomycin induced pulmonary fibrosis. RAGE knockout (KO) mice get worse fibrosis when challenged with asbestos, whereas in the bleomycin model they are largely protected against fibrosis. In the current study the role of RAGE in a mouse model of silica induced pulmonary fibrosis was investigated.

**Methodology/Principal Findings:**

Wild type (WT) and RAGE KO mice received a single intratracheal (i.t.) instillation of silica in saline or saline alone as vehicle control. Fourteen days after treatment mice were subjected to a lung mechanistic study and the lungs were lavaged and inflammatory cells, protein and TGF-β levels in lavage fluid determined. Lungs were subsequently either fixed for histology or excised for biochemical assessment of fibrosis and determination of RAGE protein- and mRNA levels. There was no difference in the inflammatory response or degree of fibrosis (hydroxyproline levels) in the lungs between WT and RAGE KO mice after silica injury. However, histologically the fibrotic lesions in the RAGE KO mice had a more diffuse alveolar septal fibrosis compared to the nodular fibrosis in WT mice. Furthermore, RAGE KO mice had a significantly higher histologic score, a measure of affected areas of the lung, compared to WT silica treated mice. A lung mechanistic study revealed a significant decrease in lung function after silica compared to control, but no difference between WT and RAGE KO. While a dose response study showed similar degrees of fibrosis after silica treatment in the two strains, the RAGE KO mice had some differences in the inflammatory response compared to WT mice.

**Conclusions/Significance:**

Aside from the difference in the fibrotic pattern, these studies showed no indicators of RAGE having an effect on the severity of pulmonary fibrosis following silica injury.

## Introduction

Pulmonary fibrosis can have various causes and can be a debilitating progressive condition with a poor prognosis. Fibrosis can develop as a response to inhaled fibrogenic fibers or particles, such as asbestos fibers or silica particles [1], [2]. It can also develop as a side effect of treatment with chemotherapeutic agents or be idiopathic (IPF) with no identifiable etiology [3], [4]. Although silicosis is 100% preventable by proper personal protection, it is still a widespread disease. In the United States the annual number of deaths with silicosis as the underlying or contributing cause has decreased from over 1,100 back in 1968 to under 200 in 2004 [5]. In contrast, China recorded 500,000 cases of silicosis in the period 1991–1995 with an incidence of around 6,000 per year with more than 24,000 deaths annually [6].

A number of murine models are utilized to study IPF and other human forms of pulmonary fibrosis in order to investigate disease progression and potential therapy. Among these models are bleomycin, fiber/particle (asbestos, silica), irradiation, fluorescein isothiocyanate, and transgenic mice strains with pulmonary specific transgenes or virus targeted transgene delivery (Reviewed in [7]).

Inhaled fibers and particles such as asbestos and silica are not effectively cleared from the lung by macrophages and instead lead to chronic inflammation and fibrosis. This is different from the bleomycin model which initiates an acute single injury followed by fibrosis and then resolution [8], [9]. Silica in the lung is first encountered by alveolar macrophages, where the membrane spanning scavenger receptor MAcrophage Receptor with COllagenous structure (MARCO) is the receptor primarily responsible for recognition and internalization of silica [10]. Not only does the uptake of silica by macrophages lead to an inflammatory response by activation of the napl3 inflammasome [11], it also causes a dose dependent cytotoxicity which leads to further inflammation culminating in pulmonary fibrosis [10].

The receptor for advanced glycation end-products (RAGE) is a member of the super family of immunoglobulin receptor molecules and exists both as a membrane bound and a soluble form lacking the trans-membrane domain [12]. Among its ligands are advanced glycation end-products (AGEs) [13], high mobility group box protein 1 (HMGB1) [14], S100 [15] and amyloid β-peptides [16]. RAGE has in recent years been implicated in a number of diseases including diabetic vascular disease and renal fibrosis [17]–[19]. In contrast to most tissues/organs where RAGE expression is normally low and increases with disease, RAGE is expressed in high amounts in the normal lung and is significantly depleted in response to injuries leading to pulmonary fibrosis [20], [21]. The role of RAGE in pulmonary fibrosis is not fully understood. Recent studies have found that RAGE KO mice spontaneously get pulmonary fibrosis as they age and also get worse fibrosis than WT mice in response to asbestos injury [21]. In contrast, RAGE KO mice are largely protected against fibrosis in a bleomycin model [22]. The bleomycin study suggested that RAGE contributes to bleomycin-induced lung fibrosis through epithelial-mesenchymal transition (EMT) and profibrotic cytokine production, and they found that RAGE KO mice had reduced BALF levels of the profibrotic cytokines transforming growth factor-β (TGF-β) and platelet-derived growth factor (PDGF) which are both RAGE inducible. Furthermore, they found that while WT primary alveolar type II epithelial cells were able to undergo EMT when stimulated with HMGB1, cells from RAGE KO mice were not, suggesting that RAGE may be involved in EMT.

In this paper data is presented from an investigation of the role of RAGE in a third model using silica to induce pulmonary fibrosis.

## Materials and Methods

### Ethics Statement

All animal experiments were reviewed and approved by the Institutional Animal Care and Use Committee at the University of Pittsburgh. Animals were given free access to food and water and were cared for according to guidelines set by the American Association for Laboratory Animal Care.

### Mouse Treatments

The RAGE KO strain was generated on a C57BL/6 background [23]. WT mice were purchased from Taconic (Germantown, NY).

Eight to 10 week old mice were anesthetized with Isoflurane (Baxter Healthcare Corporation, IL) and then received a single 70 µL i.t. instillation of either silica solution (0.2, 1, or 5 mg/mouse) or 0.9% saline as a vehicle control. Prior to instillation the 5 µm silica particles (a gift from Andy Ghio, Environmental Protection Agency) were baked at 180°C for 6 hrs. Mice were weighed every day to monitor weight loss/gain. Male mice were used for all experiments except for the dose response study.

### Determination of Respiratory Mechanics

Fourteen days after injury animals were anesthetized with 60 mg/kg pentobarbital sodium intraperitoneally (Ovation Pharmaceuticals Inc., Deerfield, IL), a tracheostomy was performed, and they were attached to a ventilator. Following 5 minutes of stabilization, every animal received deep lung inflation to 30 cm H_2_O distending pressure to ensure uniform lung recruitment, and normalization to the same pre-assessment volume history. Readings were obtained at a positive end-expiration pressure of 0 cm H_2_O in triplicate. After 2 minutes, a second set of baseline recordings were obtained without preceding deep lung inflation.

Physiological recordings were performed using a computer-controlled ventilator (flexiVent, Scientific Respiratory Equipment Inc, QC, Canada). Mice were ventilated with a tidal volume of 10 mL/kg at a respiratory rate of 200 breaths/min. Lung mechanics were measured at specific intervals by triggering the ventilator to deliver a custom input flow wave specifically developed to characterize murine lung impedance (i.e. the ratio of pressure to flow) over the physiological range of breathing frequencies (20 to 200 breaths/min).

### Inflammatory Cells

Fourteen, 21 or 28 days after treatment mice were sacrificed by an overdose of sodium pentobarbital (Ovation Pharmaceuticals Inc.) and the lungs were lavaged with a single instillation of 800 µL normal saline. Recovery of bronchoalveolar lavage fluid (BALF) was consistently above 75%. BALF cells were counted using a Z1 Coulter Particle Counter (Beckman Coulter Inc., Fullerton, CA) and 30,000 cells were transferred to glass slides using a Shandon Cytospin 4 (Thermo Electron Corporation, Pittsburgh, PA) at 750 rpm for 5 min. After 2 days of drying, cells were stained using the Diff-Quick stain (Dade Behring Inc., DE), and macrophages, neutrophils and lymphocytes were counted (200 cells).

### Protein Concentration in BALF

Protein concentration in undiluted BALF was measured by a standard endpoint measurement at 495 nm using Bradford reagent with a bovine serum albumin standard curve (Thermo Scientific, Rockford, IL).

### Hydroxyproline Assay

Collagen deposition was measured by hydroxyproline assay as previously described [24]. Briefly, dried lungs were acid hydrolyzed in 6 N HCl at 110°C under nitrogen gas. After evaporation of HCl, samples were resuspended in PBS and incubated in a 60°C water bath. Following three consecutive high speed centrifugations a 40x dilution in PBS of the supernatant was oxidized with chloramine-T and the reaction was stopped with perchloric acid. Finally, *p*-dimethylaminobenzaldehyde was added and samples were analyzed spectrophotometrically at 557 nm. Hydroxyproline content per lung was calculated from a hydroxyproline standard curve.

### Histology and Histologic Scoring

Lungs used for histology were inflation fixed with 800 µL 10% formalin for 8 min and paraffin embedded. Five µm sections were H&E stained and fibrosis was evaluated under 400× magnification by a pathologist, blinded to the nature of the mice, scoring each field of the entire lung section according to the following criteria; 0: no fibrosis, 1: 0 to 25%, 2: 25 to 50%, 3: 50 to 75% and 4: 75 to 100%, as previously described [21], [25].

### Lung Homogenate and Quantitative Real-Time PCR (qRT-PCR) Analysis

Lungs were perfused with 10 mL normal saline, excised and flash frozen in liquid nitrogen before being stored at −80°C until use. Each lung was used to get both protein and mRNA by pulverizing in liquid nitrogen as described elsewhere [26]. mRNA was extracted from 100 mg of lung powder using the RNeasy Mini Kit (Qiagen, Valencia, CA) to obtain RNA with an A260/A280 ≥1.9. One µg RNA was reverse transcriped to cDNA and real time PCR was performed on a 7300 Real-Time PCR System (Applied Biosystems, Foster City, CA) as previously described (25) using mouse GAPDH (Mm99999915_g1) and mouse β-actin (Mm00607939_s1) as endogenous controls and the RAGE probe (Mm00545815_m1) as the target gene (Applied Biosystems). Relative quantity was calculated based on the ΔΔCt method (26).

Total protein was extracted from the rest of the lung powder in CHAPS buffer as previously described [27]. Protein concentration was measured as described above for BALF and 5 µg of total protein was separated by SDS-PAGE as described below for western blotting.

### Western Blotting

SDS-PAGE was performed with 5–15% gradient gels using the glycine/2-amino-2-methyl-1,3-propanediol/HCl system as previously described [28]. Proteins were transferred onto a PVDF membrane (Millipore, Bedford, MA) and the membrane was blocked in 5% milk in PBS-T.

The membrane was incubated with a 1∶5,000 dilution of primary antibody against RAGE generated as previously described [29] or a 1∶1,000 dilution of rabbit-anti HMGB1 (Abcam, Cambridge, MA). After washing, the membrane was incubated with a HRP-conjugated donkey-anti-rabbit antibody (GE Healthcare, Buckinghamshire, UK). The membrane was developed and visualized using the enhanced chemiluminescent plus reagent (GE Healthcare) and a KODAK GelLogic 2200 Imaging system (Carestream Health, Rochester, NY). Loading control was performed by normalizing band intensity to β-actin (Sigma-Aldrich, St. Louis, MO). For protein loading control of BALF samples PVDF membranes were stained with ponceau S stain.

### ELISA for TGF-β Concentration in BALF

Active and total TGF-β in BALF was measured in undiluted BALF using the mouse TGF-β Duoset ELISA Development kit (R&D systems Inc., Minneapolis, MN) according to manufactures instructions for cell media.

### Statistical Analysis

Data were analyzed using GraphPad Prism 5.0 (GraphPad Software Inc., La Jolla, CA). Experiments involving both WT and RAGE KO (4 groups) were analyzed by two-way analysis of variance with a Bonferroni post-test. Data with one variable were analyzed by a Mann Whitney test. All values are means (±SEM). * p<0.05 was considered significant.

## Results

### RAGE Protein and RAGE mRNA Are Down Regulated in Lungs with Silicosis

**Figure 1:**
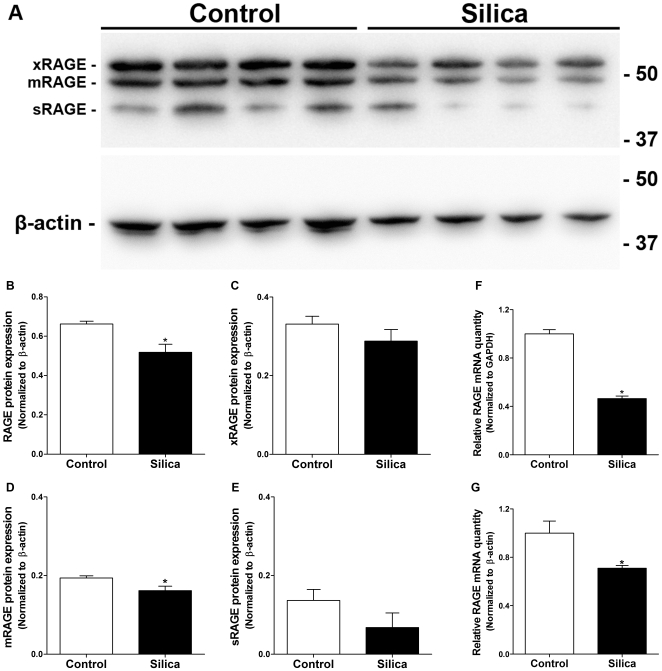
RAGE protein and mRNA levels are decreased after silica injury. (A) Western blot of total protein in lung homogenate of lungs from control and silica treated WT mice, 5 µg of protein were loaded per lane. In addition to mRAGE and sRAGE a third isoform of RAGE, recently termed xRAGE [40], was also detected by western blot. (B) Protein levels of total RAGE normalized to β-actin revealed a significant decrease in expression after silica injury. (C–E) When split into the different isoforms only mRAGE showed a significant decrease in protein, while both xRAGE and sRAGE had a trend towards a decrease. (F–G) RAGE mRNA levels were significantly decreased after silica injury supporting the observation of the protein levels. This was significant when normalizing to both of the endogenous controls GAPDH and β-actin. Mann Whitney test (*n = 4–5 per group*). * p<0.05 silica vs. control.

Membrane RAGE is highly expressed on epithelial type I cells in the lung and consequently has been suggested to be a marker of loss of epithelial type I cells [30]. During inflammation and development of fibrotic areas in the lung, type I epithelial cells are injured and lost which should lead to decreased levels of membrane RAGE in the lung. RAGE protein levels in total lung homogenate 14 days after silica challenge was investigated (Figure 1A–E). Consistent with the above hypothesis, a significant decrease of total RAGE was observed. In addition, while the individual isoforms xRAGE and sRAGE only trended towards a decrease, mRAGE decreased significantly after silica injury. Furthermore, RAGE mRNA levels were significantly decreased in silica injured lungs (Figure 1F–G), which is consistent with the decrease in protein levels.

### Hydroxyproline Levels in the Lungs after Injury

**Figure 2:**
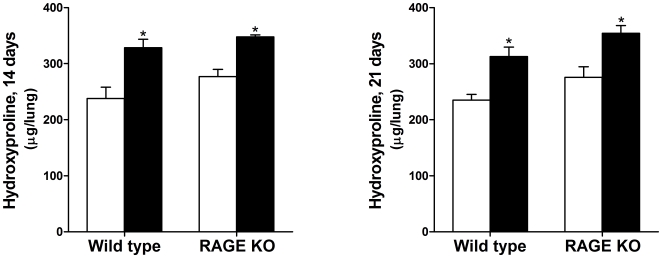
Hydroxyproline levels in WT and RAGE KO lungs were significantly higher in silica treated (closed bars) compared to saline controls (open bars). There were no significant differences between WT and RAGE KO hydroxyproline levels in the silica injured mice after both 14- and 21 days. Data were analyzed using 2-way ANOVA with a Bonferroni post-test and are means (±SEM) (*n = 5–6 per group*). * p<0.05 silica vs. control.

Only a few macromolecules such as the scleroproteins collagen and elastin contain hydroxyproline in significant amounts, with collagen the most abundant source [24]. Thus, hydroxyproline levels directly correlate with levels of collagen deposition in the lung tissue and is used as a measure of fibrosis after silica injury. While hydroxyproline levels in the lungs of silica treated WT and RAGE KO mice were both significantly higher than control treated lungs 14- and 21 days after silica challenge, there were no significant differences between the two strains (Figure 2).

### RAGE KO Mice Display a Different Histologic Pattern of Fibrosis Compared to WT Mice

**Figure 3:**
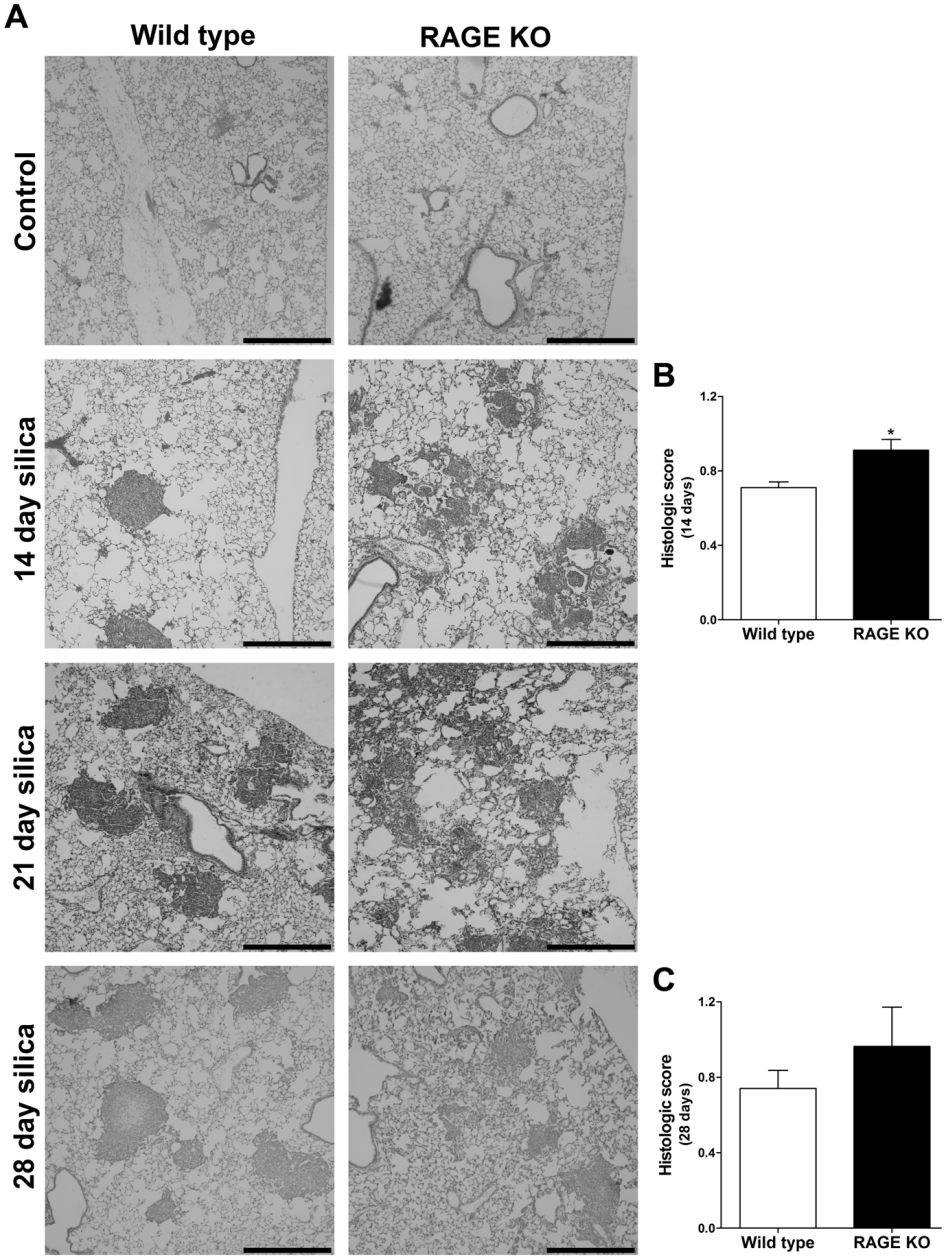
RAGE KO mice have a different fibrotic pattern than WT mice. Lungs were fixed in 10% formalin and paraffin embedded. After H&E staining the lungs were inspected by microscopy (A) and scored according to the degree of fibrosis in each high power field (B and C). When comparing the silica treated WT and RAGE KO lung sections, it is evident that there is a marked difference in the fibrotic pattern among the two strains at both the 14-, 21-, and to a lesser extend the 28 day time point. WT mice have characteristic nodular shaped fibrotic regions, whereas RAGE KO mice develop less dense nodules with a more diffuse alveolar septal fibrosis. Silica treatment resulted in a significant increase in the histologic score for both WT and RAGE KO at the 14- and 28 day time points, and RAGE KO mice had a significantly higher score than WT mice after 14 days (B). After 28 days there was a trend towards what was seen after 14 days (C). Both WT and RAGE KO control treated mice had no fibrosis and therefore a score of 0 throughout (Data not shown). Data are means (±SEM) analyzed by 2-way ANOVA with a Bonferroni post-test. Black scale bars represent 500 µm. (*n = 5–6 per group for 14 day time point and n = 3 per group for 28 day time point*). * p<0.05 RAGE KO vs. WT.

H&E staining of lungs 14-, 21-, and 28 days post treatment revealed a different histologic pattern of fibrosis among the two mouse strains (Figure 3A). While the WT mice developed characteristic nodular shaped fibrotic areas associated with silica induced fibrosis, the RAGE KO mice developed nodular lesions with a more diffuse fibrosis which extends from the nodules into the alveolar septa. In addition, histologic scoring revealed a significantly higher degree of affected area in the silica treated RAGE KO mice compared to WT mice after 14 days (Figure 3B), but not after 28 days (Figure 3C). This may suggest that absence of RAGE delays the coalescence of the fibrotic nodules.

### Analysis of White Blood Cells and Protein in BALF

**Figure 4:**
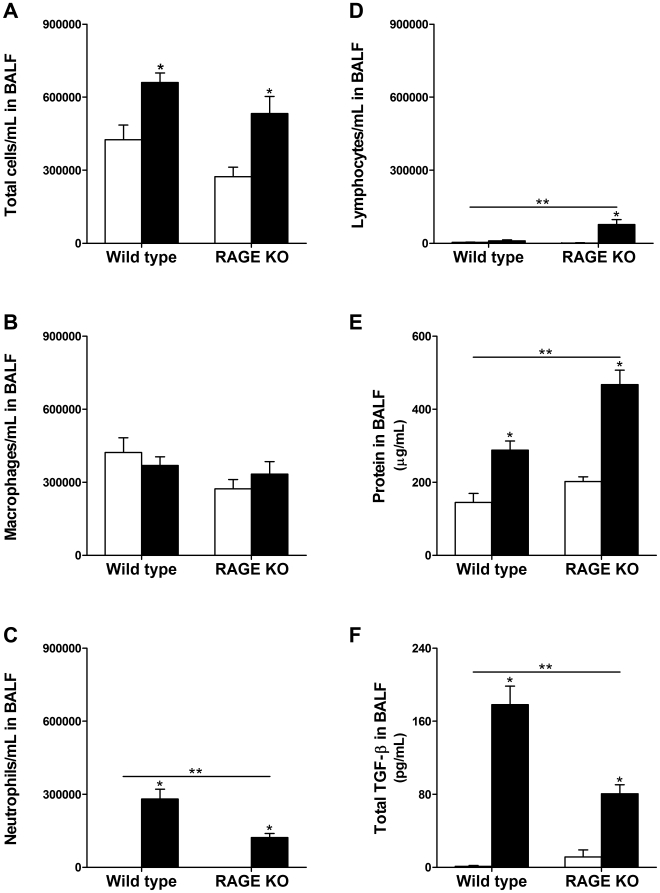
Analysis of BALF specimens 14 days after treatment. Lungs were lavaged with 800 µL saline and cells were counted in triplicate. Thirty thousand cells were transferred to a glass slide using a cytospin and stained. Two hundred cells were counted to determine the percentage of macrophages, lymphocytes and neutrophils. (A) Both silica treated WT and RAGE KO (closed bars) had significantly more total cells per mL in the BALF compared to control treated mice (open bars). There was not a significant difference between WT and RAGE KO mice. However, while there was no difference in number of macrophages (B), RAGE KO mice had significantly less neutrophils but more lymphocytes than WT mice treated with silica (C–D). (E) Protein concentration in the BALF was used as a measure of lung permeability and hence lung injury. There was a significant increase in protein concentration in silica treated BALF from both WT and RAGE KO over the controls. In addition, silica treated RAGE KO mice had significantly higher protein concentration in the BALF compared to WT mice. (F) Total TGF-β level in BALF was significantly lower in RAGE KO compared to WT silica treated mice. No active TGF-β was detected in BALF samples. Data are means (±SEM) analyzed by 2-way ANOVA with a Bonferroni post-test. Asterisks above error bars represent comparison to the control treated of the same strain. Asterisks above line represent an interaction and hence a difference between WT and RAGE KO mice. (*n = 7–9 per group*). * p<0.05 silica vs. control, ** p<0.05 WT vs. RAGE KO.

**Figure 5:**
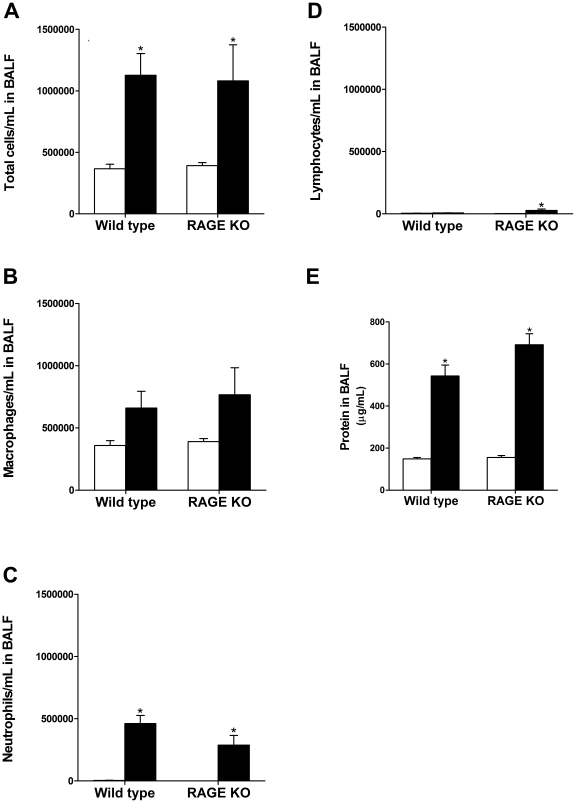
Analysis of BALF specimens 21 days after treatment. (A) Both silica treated WT and RAGE KO (closed bars) had significantly more total cells per mL in the BALF compared to control treated mice (open bars). There was not a significant difference between WT and RAGE KO mice. Furthermore, the number of macrophages in both WT and RAGE KO BALF was not significantly increased over control treated mice (B). While the level of neutrophils increased significantly with silica treatment compared to controls for both strains (C), only RAGE KO mice had a significant increase in lymphocytes after silica treatment (D). (E) Protein concentration in the BALF also increased significantly with treatment, but there was no difference between the two strains. Data are means (±SEM) analyzed by 2-way ANOVA with a Bonferroni post-test. (*n = 6–7 per group*). * p<0.05 silica vs. control.

Total cells/mL in the BALF was significantly increased 14- and 21 days after silica challenge for both WT and RAGE KO mice (Figure 4A and 5A). However, there were no differences between the two strains. Analysis of leukocytes in the BALF showed that in both WT and RAGE KO control treated mice the main cell type found was the macrophage with a very small amount of neutrophils and lymphocytes (Figure 4B–D and 5B–D). Fourteen days after silica treatment there was an increase in neutrophils compared to control treatment for both WT and RAGE KO. Lymphocytes on the other hand were only significantly up regulated in RAGE KO mice after silica injury. Furthermore, there was a significantly higher increase in neutrophils in the WT compared to RAGE KO. In contrast, RAGE KO mice had significantly more lymphocytes after injury than WT mice. Twenty one days after silica treatment there was no longer a significant difference in the neutrophil population between control and silica treated mice (Figure 5C), and only the RAGE KO mice had a significant increase in lymphocytes compared to control (Figure 5D).

Protein concentration in the BALF was used as another measure of lung injury. Injury to the lung parenchyma and loss of epithelial cells leads to leakage from the vasculature of proteinaceous fluid into the lung. RAGE KO mice had significantly higher protein concentration in the BALF 14 days after silica injury compared to WT silica treated mice (Figure 4E), but 21 days after injury there was only a trend towards more protein in the BALF of the RAGE KO mice (Figure 5E).

### Effect of Knock Out of RAGE on Total TGF-β Concentration in BALF

TGF-β is an important growth factor involved in the development of pulmonary fibrosis (Reviewed in [31], [32]). The level of total TGF-β in the BALF of both control and silica treated WT and RAGE KO mice 14 days after treatment was assayed by ELISA (Figure 4F). Silica treated RAGE KO mice had a significantly lower concentration of TGF-β in the BALF compared to silica treated WT mice. This is consistent with a previously report that TGF-β levels were lower in BALF from bleomycin treated RAGE KO compared to WT mice [22].

### Western Blot of Bronchoalveolar Lavage Fluid for Soluble RAGE and HMGB1

**Figure 6:**
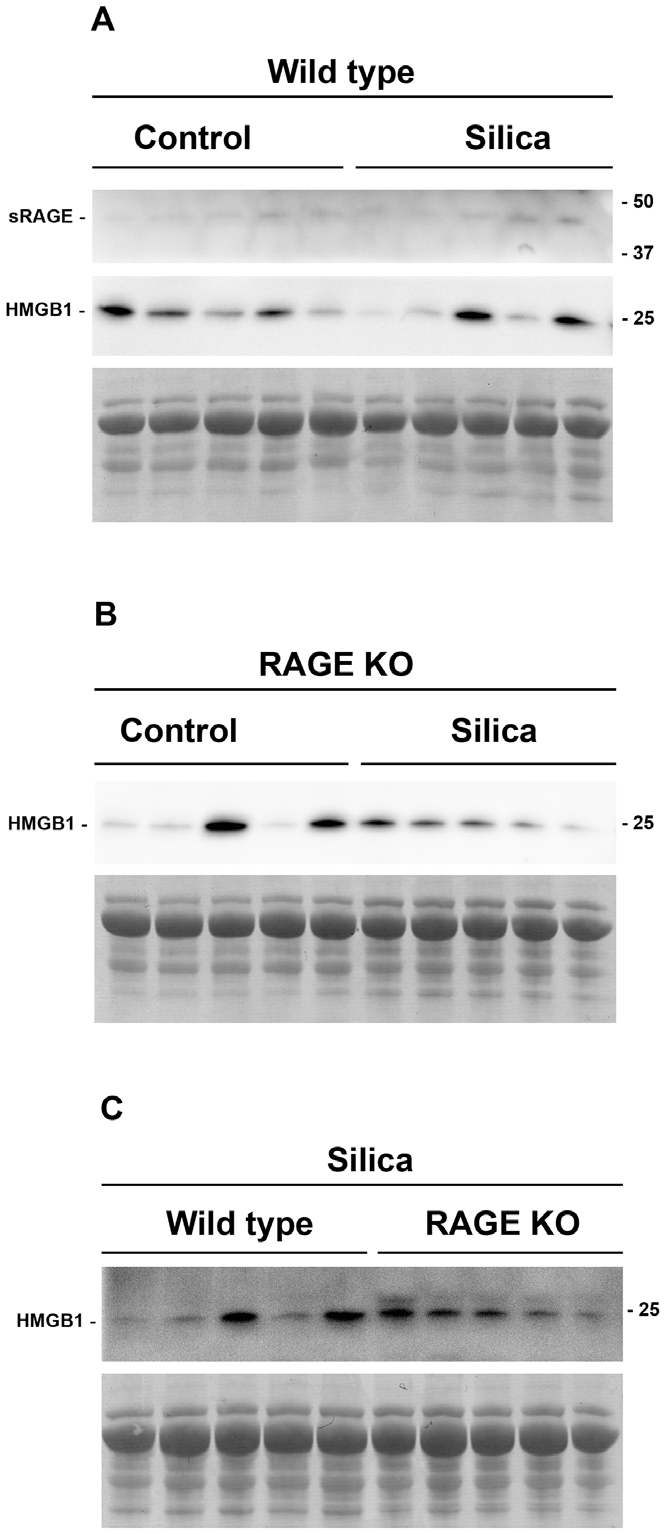
Western blot of BALF specimens for soluble RAGE and HMGB1. Equal amounts of protein were loaded in each lane of the individual gels to assure maximum loading of protein. Protein amounts were limited to the maximum amount of protein that could be loaded from the most dilute sample. Protein amounts were as follows; A, 4.4 µg; B, 6.9 µg; C, 11.2 µg. Lower panel in each section shows a section of the ponceau S stained membrane, to assure equal loading of protein. Comparison between blots is not the intension of these western blots. Densitometry analysis was performed on the soluble RAGE and HMGB1 bands, and different treatments compared within each gel by a Mann Whitney test (Data not shown). (A) Compares the two proteins without and with silica treatment in WT mice. Some very weak soluble RAGE bands are visible both in control and silica treated WT BALF. HMGB1 levels were the same in control and silica treated WT mice, and because equal protein was loaded and the fact that the protein concentration in the BALF of WT mice after silica challenge approximately doubled, the overall amount of HMBG1 in the BALF (I.e. amount per volume BALF) is actually increased, this observation was the same for RAGE KO mice (B). (C) Compares HMGB1 levels after treatment with silica in WT and RAGE KO. HMGB1 levels varied to a high degree between samples, and there was no significant difference between the two strains as determined by densitometry analysis.

BALF samples were assayed by western blot for soluble RAGE and the inflammatory cytokine HMGB1 14 days after silica challenge (Figure 6). Soluble RAGE levels have previously been shown to increase in the BALF in a model of acute lung injury [33]. Furthermore, HMGB1 has been shown to be able to induce EMT *in vitro* [22], hence increased levels of HMGB1 could potentially drive EMT and fibrosis. Soluble RAGE in the BALF was slightly increased in WT BALF after silica challenge (Figure 6A). The level of HMGB1 after silica treatment in both the WT and RAGE KO mice was also increased (Figure 6A–B). From the western blot the level appears to be the same, but equal amount of protein was loaded in each lane and since the silica treated mice had higher protein concentration in the BALF (Figure 4), an equal intensity actually indicates a higher level of HMGB1 on a per volume basis. Furthermore, there were no significant differences in the level of HMGB1 between WT and RAGE KO silica treated mice (Figure 6C).

### Lung Mechanistic Study

Fibrotic areas in the lung lead to decreased pulmonary function because the collagen deposition leads to a restriction in flexibility of the normally very elastic structure of the lung. By the use of delicate equipment such as the flexiVent system it is possible to collect information on the functional state of small lungs such as those from mice [34].

**Figure 7:**
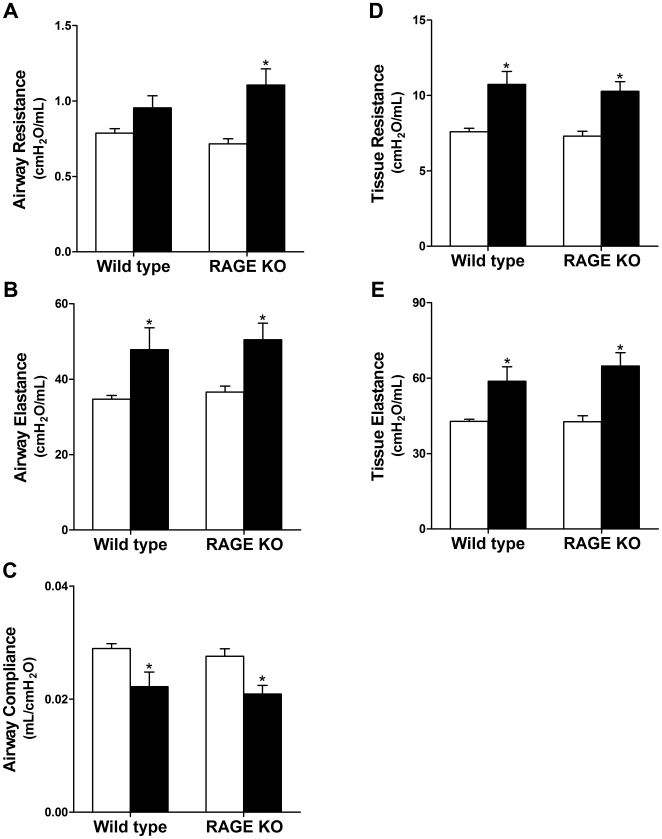
Lung mechanics after silica injury. Changes in lung mechanics were similar in WT and RAGE KO mice. Closed bars represent silica treatment and open bars vehicle control. (A) While the airway resistance in silica treated WT mice was not significantly increased over control, the RAGE KO mice treated with silica had a significant increase. All other values (B–E) were significantly changed for both WT and RAGE KO; however there were no other significant differences between WT and RAGE KO. Data are means (±SEM) analyzed by 2-way ANOVA with a Bonferroni post-test. (*n = 5–6 per group*). * p<0.05 silica vs. control.

Airway and tissue resistance, -compliance and -elastance were measured (Figure 7). We hypothesized that the more diffuse development of fibrosis in RAGE KO mice would lead to a more impaired pulmonary function than the WT mice. While silica treated WT and RAGE KO mice had a significant decrease in pulmonary function as measured by airway resistance, -compliance, and -elastance, and tissue resistance and -elastance, over the saline treated controls, there was no statistical significant difference between WT and RAGE KO mice. Airway resistance was only significantly increased in RAGE KO silica mice compared to control, and not in WT silica vs. control mice (Figure 7A).

### Dose Response in Female Mice

**Figure 8:**
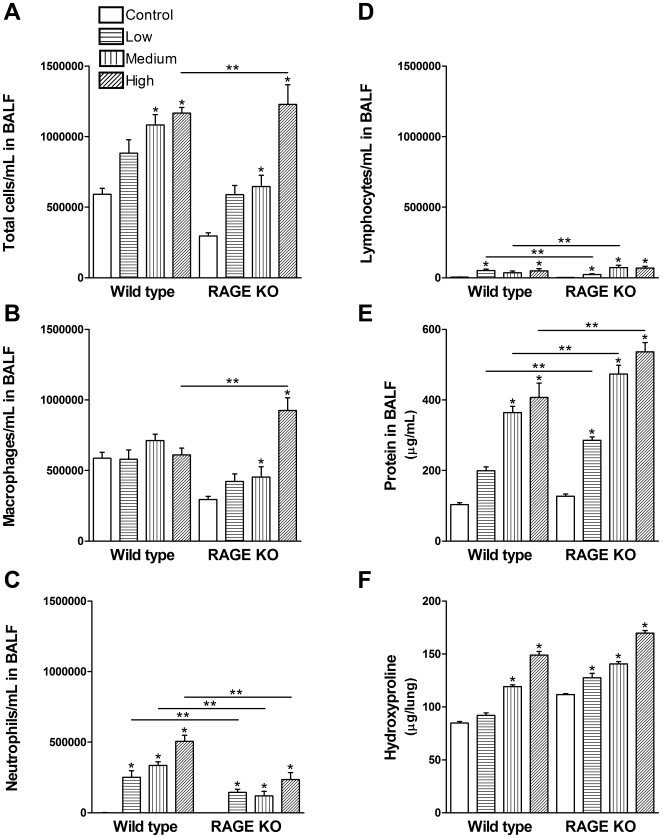
Dose response to silica. (A) The high dose of silica resulted in a significantly higher increase in total cells/mL in the BALF of RAGE KO mice compared to WT mice. (B) RAGE KO mice had more macrophages than WT at the high dose of silica. (C) Neutrophil levels were significantly lower in RAGE KO mice compared to WT mice for all doses of silica. (D) Lymphocyte levels were different among the two strains at the two lower doses of silica. (E) RAGE KO mice had a significant higher level of protein in the BALF after all doses of silica compared to WT mice. (F) Hydroxyproline assay showed no difference in collagen deposition between the two strains. Low; 0.2 mg, medium; 1 mg, high; 5 mg silica. Data are means (±SEM) analyzed by 2-way ANOVA with a Bonferroni post-test. (*n = 6–7 per group*). * p<0.05 silica vs. control, ** p<0.05 WT vs. RAGE KO.

In order to verify that the above observations were consistent using different amounts of silica, a dose response was carried out in female mice and sacrificed 21 days after silica challenge. Doses of 0.2 mg, 1 mg and 5 mg silica per mouse were used. Analysis of BALF cells, protein concentration in BALF and hydroxyproline levels in whole lungs were used to assess inflammation and fibrosis. Only the high dose of silica led to a significant higher level of cells in the BALF from RAGE KO mice compared to WT mice (Figure 8A), which were mainly macrophages (Figure 8B). In addition, RAGE KO mice had less neutrophils with silica treatment compared to wild type mice for all doses of silica (Figure 8C) and slightly different lymphocyte infiltration at the low and medium dose of silica (Figure 8D). Furthermore, protein concentration in the BALF was significantly higher in the RAGE KO mice at all doses of silica compared to WT mice (Figure 8E). However, there were no differences in hydroxyproline levels between the two strains at any of the 3 different doses of silica (Figure 8F). H&E staining of fixed lung sections from the different doses showed no signs of fibrosis at the low dose, very mild changes in the medium dose, and a similar extent and pattern of fibrosis as indicated in figure 3 for the high dose of 5 mg silica per mouse (Data not illustrated).

## Discussion

The role of RAGE has previously been studied in an asbestos and a bleomycin model of pulmonary fibrosis [21], [22]. Notably, the absence of RAGE was found to lead to spontaneous fibrosis with aging and increased fibrosis in response to asbestos injury [21]. In contrast, RAGE KO mice were found to be largely protected against bleomycin induced fibrosis [22]. Here, the role of RAGE in a silica model of pulmonary fibrosis was studied. Injury led to a decrease in RAGE mRNA levels and a decrease in total RAGE protein in total lung homogenates. This is consistent with findings in both the bleomycin model and asbestos model as well as in lungs from human IPF patients [20]–[22]. The decrease in membrane RAGE protein is likely to be a direct effect of loss of type I epithelial cells and may also be due to cleavage of membrane RAGE by proteases to form soluble RAGE [35], [36]. The loss of soluble RAGE is more likely to be an indicator of tissue damage and damage to the extracellular matrix, but may be due to decreased production from injured epithelial cells. Previous studies have shown that shedding of syndecan-1, a heparin sulfate proteoglycan highly abundant in the extracellular matrix, occurs in models of pulmonary fibrosis [37]. Since soluble RAGE has high affinity to heparin sulfate, collagen and other extracellular matrix proteins [29], [36], [38], it is likely that damage to those structures will result in release of bound soluble RAGE. In fact, soluble RAGE has been shown to accumulate in the BALF after acute lung injury [33]. However, barely any soluble RAGE was detected in the BALF of WT mice both before and after silica injury. This may be due to clearance of soluble RAGE from the BALF after the initial injury.

The silica model of pulmonary fibrosis is more closely related to the asbestos model than the bleomycin model, in that the silica particles are relatively resistant to clearance from the lung and lead to inflammation and fibrosis around the silica particles. This is visualized by the characteristic silicotic nodules (Figure 3, wild type silica). In the silica model we hypothesized the outcome to resemble what takes place in the asbestos model, since they are both fibers/particles that persist in the lung. Furthermore, RAGE/collagen IV interactions have previously been shown to be important for epithelial cell spreading [38] and possibly wound healing. Based on this we hypothesized that an increase of RAGE ligands could lead to decreased re-epithelialization after injury and furthermore that RAGE KO mice would get worse fibrosis since they would have decreased ability to re-epithelialize. However, while we found no difference in the absolute degree of fibrosis between WT and RAGE KO mice treated with silica, histologically the RAGE KO mice had a more diffuse pattern of fibrosis compared to the dense nodular fibrosis in the WT mice. Pulmonary function studies also showed a trend of increased airway resistance in the RAGE KO compared to WT after silica injury. The RAGE/collagen IV interaction might explain why RAGE expressing WT mice are able to restrict the spreading of fibrosis more effectively than RAGE KO mice. The interaction may function to control the cell spreading even during wound healing, and hence the fibrosis develops as nodules with clearly defined edges, whereas RAGE KO mice are not able to control the spreading of epithelial cells and therefore develop a more diffuse pattern of fibrosis. Another possibility could be that macrophages phagocytozing silica particles subsequently migrate towards each other in order to condense the silica particles and limit the extent of fibrosis. It could be that RAGE KO macrophages have a changed ability to perform this task.

TGF-β levels in BALF samples of silica treated RAGE KO mice were significantly lower than in WT mice. This finding may explain why the two strains get similar amount of fibrosis. As previously mentioned RAGE KO mice get more fibrosis in an asbestos model, while in a bleomycin model they are largely protected [21], [22]. If RAGE KO mice are actually more susceptible to developing fibrosis when challenged i.t. with a particular/fibrillar agent, but at the same time have less TGF-β, then the overall amount of fibrosis may end up being the same.

A dose response study with 3 different amounts of silica showed similar degrees of fibrosis, but some differences in inflammatory cell population between RAGE KO and WT mice. Furthermore, protein leakage into the lung was significantly higher in RAGE KO mice than in WT mice, indicating that the integrity of the alveolar wall is more fragile/susceptible to damage in RAGE KO mice. Taken together this indicates that RAGE may be involved in the inflammatory response and adherence of epithelial cells, but not in the subsequent development of fibrosis after silica induced lung damage. The RAGE KO mice used in this study are constitutive global RAGE KO mice. It is possible that the constitutive RAGE KO mice may have up- or down regulated proteins as an adaptive response to the loss of pulmonary RAGE expression. RAGE may also be involved in the regulation of other proteins whose expression could be dramatically altered by the knockout of RAGE. Notably, a recent study using inducible EC-SOD KO mice showed that while constitutive EC-SOD KO mice were viable and had a phenotype resembling WT mice, the conditional EC-SOD KO mice died rapidly with symptoms resembling acute lung injury after inducing the knockout of EC-SDO [39]. Further studies will be necessary to study potential differentially regulated proteins between WT and RAGE KO mice. In addition, an inducible RAGE KO mouse strain may help shed light on the inflammatory function of RAGE and its involvement in lung pathology where this protein is so highly expressed.

In summary, except for the altered histologic pattern of fibrosis, there is no data to suggest that RAGE expression alters the degree of fibrosis using a silica induced model of pulmonary fibrosis. RAGE does appear to be a strong marker of epithelial cell injury in that both a decrease in pulmonary protein levels of RAGE and a decrease in RAGE mRNA levels are seen in response to fibrotic injury. The markedly different responses of RAGE KO mice to different models of pulmonary fibrosis suggest that RAGE does play a role in the pathogenesis of pulmonary fibrosis, but its role depends on the injury eliciting the fibrotic response. Further investigation is necessary in order to fully understand the mechanisms RAGE plays in pulmonary fibrosis and how to best exploit RAGE biology to intervene in the fibrotic response.
